# Medical Witnesses' Bill

**Published:** 1836-10

**Authors:** 


### MEDICAL WITNESSES' BILL.

In giving a copy of this excellent Bill, we feel bound to express our acknowledgments, in common with the profession generally, to the author of it, Mr. Wakley, M.P. for Finsbury. It is certainly matter of regret that there are not more members in the House of Commons qualified and disposed to watch over the interests of the medical profession. We yet hope to see the defect satisfactorily supplied.

*An Act to provide for the Attendance and Remuneration of Medical Witnesses at Coroner's Inquests.* [August 18th, 1836.]

I. Whereas it is expedient to provide for the attendance of medical witnesses at coroners' inquests, also remuneration for such attendance, and for the performance of post-mortem examinations at such inquests: Be it therefore enacted, . . . that, from and after the passing of this act, whenever, upon the summoning or holding of any coroner's inquest, it shall appear to the coroner that the deceased person was attended at his death, or during his last illness, by any legally qualified medical practitioner, it shall be lawful for the coroner to issue his order . . . for the attendance of such practitioner as a witness at such inquest; and if it shall appear to the coroner that the deceased person was not attended at or immediately before his death by any legally qualified medical practitioner, it shall be lawful for the coroner to issue such order for the attendance of any legally qualified medical practitioner, being at the time in actual practice in or near the place where the death has happened; and it shall be lawful for the coroner, either in his order for the attendance of the medical witness, or at any time between the issuing of such order and the termination of the inquest, to direct the performance of a post-mortem examination, with or without an analysis of the contents of the stomach or intestines, by the medical witness or witnesses who may be summoned to attend at any inquest, provided that if any person shall state upon oath before the coroner that, in his or her belief, the death of the deceased individual was caused partly or entirely by the improper or negligent treatment of any medical practitioner or other person, such medical practitioner or other person shall not be allowed to perform or assist at the post-mortem examination of the deceased.

II. And be it further enacted, That whenever it shall appear to the greater number of the jurymen sitting at any coroner's inquest, that the cause of death has not been satisfactorily explained by the evidence of the medical practitioner or other witness or witnesses who may be examined in the first instance, such greater number of the jurymen are hereby authorised and empowered to name to the coroner, in writing, any other legally qualified medical practitioner or practitioners, and to require the coroner to issue his order . . . for the attendance of such last-mentioned medical practitioner or practitioners as a witness or witnesses, and for the performance of a post-mortem examination, with or without an analysis of the contents of the stomach or intestines, whether such an examination has been performed before or not; and if the coroner, having been thereunto required, shall refuse to issue such order, he shall be deemed guilty of a misdemeanour, and shall be punishable in like manner as if the same were a misdemeanour at common law.

III. And be it further enacted, That when any legally qualified medical practitioner has attended upon any coroner's inquest, in obedience to any such order as aforesaid of the coroner, the said practitioner shall for such attendance at any inquest in Great Britain be entitled to receive such remuneration or fee as is mentioned in the table marked (B.) in the schedule hereunto annexed; and for any inquest held in Ireland, the said practitioner shall be paid in the manner provided by the laws in force in that part of the United Kingdom; and the coroner is hereby required and commanded to make . . . his order for the payment of such remuneration or fee, when the inquest shall be held in Great Britain; and such order may be addressed and directed to the churchwardens and overseers of the parish or place in which the death has happened; and such churchwardens and overseers, or any one of them, is and are hereby required and commanded to pay the sum of money mentioned in such order of the coroner to the medical witness therein mentioned, out of the funds collected for the relief of the poor of the said place.

IV. Provided nevertheless, and be it further enacted, That no order of payment shall be given, or fee or remuneration paid, to any medical practitioner for the performance of any post-mortem examination which may be instituted without the previous direction of the coroner.

V. Provided also, and be it further enacted, That when any inquest shall be holden on the body of any person who has died in any public hospital or infirmary, or any building or place belonging thereto, or used for the reception of the patients thereof, or who has died in any county or other lunatic asylum, or in any public infirmary or other public medical institution, whether the same be supported by endowments or by voluntary subscriptions, then and in such case nothing herein contained shall be construed to entitle the medical officer whose duty it may have been to attend the deceased person as a medical officer of such institution as aforesaid to the fees or remuneration herein provided.

VI. And be it further enacted, That where any order for the attendance of any medical practitioner as aforesaid shall have been personally served upon such practitioner, or where any such order not personally served shall have been received by any medical practitioner in sufficient time for him to have obeyed such order, or where any such order has been served at the residence of any medical practitioner; and in every case where any medical practitioner has not obeyed such order, he shall, for such neglect or disobedience, forfeit the sum of five pounds sterling, upon complaint thereof made by the coroner or any two of the jury before any two justices having jurisdiction in the parish or place where the inquest under which the order issued was held, or in the parish where such medical practitioner resides; and such two justices are hereby required, upon such complaint, to proceed to the hearing and adjudication of such complaint, and if such medical practitioner shall not show to the said justices a good and sufficient cause for not having obeyed such order, to enforce the said penalty by distress and sale of the offender's goods, as they are empowered to proceed by an act of parliament for any other penalty or forfeiture.

VII. And be it enacted, That nothing in this act contained shall extend to Scotland.

(B.) *Table of Fees.*

1. To every legally qualified medical practitioner, for attending to give evidence under the provisions of this act, at any coroner's inquest, whereat no post-mortem examination has been made by such practitioner, the fee or remuneration shall be one guinea.
2. For the making of a post-mortem examination of the body of the deceased, either with or without an analysis of the contents of the stomach or intestines, and for attending to give evidence thereon, the fee or remuneration shall be two guineas.

---

UNIVERSITY OF EDINBURGH. PRIZES TO BE GIVEN BY THE MEDICAL FACULTY.

